# Distinct Genetic Signatures of Cortical and Subcortical Regions Associated with Human Memory

**DOI:** 10.1523/ENEURO.0283-19.2019

**Published:** 2019-12-13

**Authors:** Pin Kwang Tan, Egor Ananyev, Po-Jang Hsieh

**Affiliations:** 1 The N.1 Institute of Health, National University of Singapore, Singapore, Singapore 117456; 2 Department of Psychology, Nanyang Technological University, Singapore, Singapore 639798; 3 Department of Psychology, National Taiwan University, Taipei City, Taiwan 10617

**Keywords:** cognition, cortical, genetic, human, memory, neuroimaging

## Abstract

Despite the discovery of gene variants linked to memory performance, understanding the genetic basis of adult human memory remains a challenge. Here, we devised an unsupervised framework that relies on spatial correlations between human transcriptome data and functional neuroimaging maps to uncover the genetic signatures of memory in functionally-defined cortical and subcortical memory regions. Results were validated with animal literature and showed that our framework is highly effective in identifying memory-related processes and genes compared to a control cognitive function. Genes preferentially expressed in cortical memory regions are linked to memory-related processes such as immune and epigenetic regulation. Genes expressed in subcortical memory regions are associated with neurogenesis and glial cell differentiation. Genes expressed in both cortical and subcortical memory areas are involved in the regulation of transcription, synaptic plasticity, and glutamate receptor signaling. Furthermore, distinct memory-associated genes such as *PRKCD* and *CDK5* are linked to cortical and subcortical regions, respectively. Thus, cortical and subcortical memory regions exhibit distinct genetic signatures that potentially reflect functional differences in health and disease, and nominates gene candidates for future experimental investigations.

## Abstract (precis)

Despite the discovery of gene variants linked to memory performance, understanding the genetic basis of adult human memory remains a challenge. Here, we devised an unsupervised framework that relies on spatial correlations between human transcriptome data and functional neuroimaging maps to uncover the genetic signatures of memory in functionally-defined cortical and subcortical memory regions.

## Significance Statement

The anatomic and functional aspects of human memory are well characterized, but its biological mechanisms are poorly understood. Here, to uncover genetic signatures associated with human memory function, we analyzed spatial correlations between micro-scale gene expression and macro-scale neuroimaging maps to derive memory-related biological processes and genes in an unsupervised manner. We found the gene signatures of cortical and subcortical memory to be largely distinct and are associated with memory. We identified less characterized memory-associated genes as well. Furthermore, our framework demonstrated effectiveness and precision in identifying gene signatures related to memory versus another function as a control. Overall, our work provides a human-centric approach to understanding the genetics of cognition and identifies potential gene candidates for future experimental investigations.

## Introduction

Memory function is crucial for everyday life, ranging from mental arithmetic to long-term planning. Human memory function is well characterized in terms of neural correlates associated with behavior and mental disorders. Insights from fMRI and lesion studies led to an understanding of cortical and subcortical memory regions as functionally distinct areas, subsumed under the broad umbrella of memory function (LaBar and Cabeza, 2006; Squire and Wixted, 2011). Yet, despite the fact that memory ability is highly heritable, with genetic risk factors for memory disorders, the genetic signature underlying human memory remains poorly understood (Papassotiropoulos and de Quervain, 2011; Kandel et al., 2014; Freudenberg-Hua et al., 2018). Our knowledge of human memory genes is largely based on interindividual variation in genomes [e.g., genome-wide association studies (GWAS)] and the short-term temporal dynamics of memory function (Berto et al., 2017). However, there is emerging interest in using the spatial dimension of gene expression to identify genetic profiles of functional networks, by integrating human brain transcriptomes and neuroimaging maps (Yarkoni et al., 2011; Hawrylycz et al., 2012; Ritchie et al., 2018). Such approaches based on spatial expression patterns may help answer a key question: Are there genes associated with general memory regions in the adult human brain? This may provide unprecedented insight into biological processes and genes associated with human memory, and propose potential candidates for further experimental investigation.

**Figure 1.**
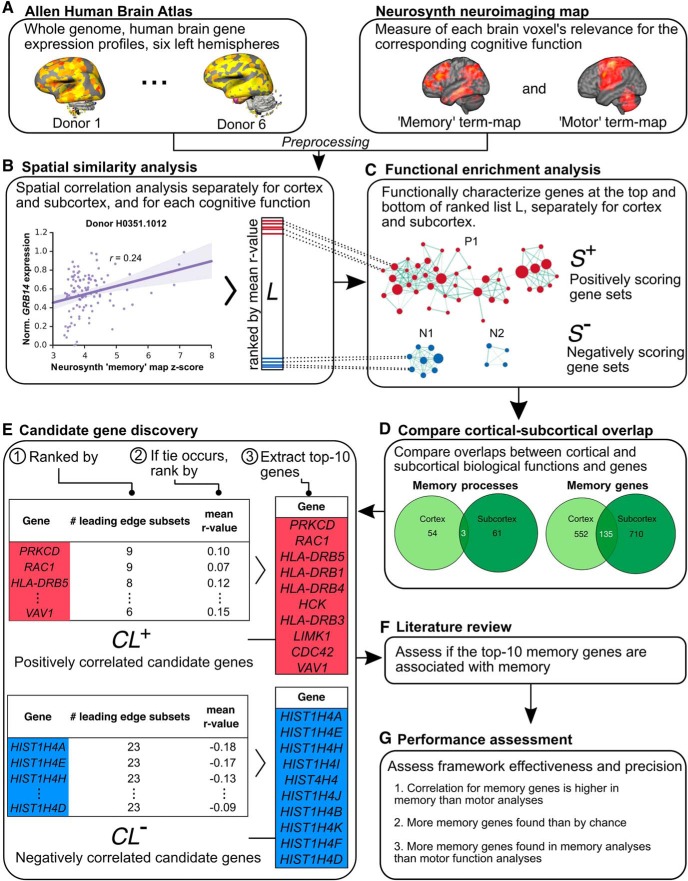
Overview of genetic signature discovery framework. ***A***, The AHBA and Neurosynth neuroimaging maps, and their preprocessing and integration into a common neuroimaging template space. ***B***, Calculation of spatial similarity between the maps separately for the cortical and subcortical regions, and for memory and motor functions, deriving a ranked gene list *L* per analysis (contains genes and mean *r* value). ***C***, Functional characterization of each *L* with biologically meaningful gene sets with GSEA Pre-ranked analysis (dotted lines connecting *L* and gene sets represent the clustering of genes into enriched gene sets), yielding positively and negatively scoring gene sets *S* ^+^ and *S* ^−^. ***D***, Assessing differences and the overlap between cortical and subcortical memory genes. ***E***, Identification of candidate genes associated with the cognitive function and brain region, operationalized as the subset of genes driving the enrichment score of the significantly enriched gene sets found using GSEA Pre-ranked analysis. This produced two candidate gene lists, *CL* ^+^ and *CL* ^−^, containing highly positively and negatively correlated genes from *S* ^+^ and *S* ^−^, respectively. ***F***, Literature review of each *CL* quantifying the genes associated with the target or control cognitive function. ***G***, Assessing framework validity and precision with each of eight *CL*s. See Extended Data Figure 1-1 for a visualization of *GRB14* gene expression in the AHBA, Extended Data Figure 1-2 for a visualization of the Neurosynth maps, and Extended Data Figure 1-3 for the cortical and subcortical regions used in the spatial correlation analysis.

To identify such adult human genes associated with general memory, we rely on a spatial correlation method (Fox et al., 2014). The method identifies cognition-associated genes that have a high spatial correlation between its gene expression and a neuroimaging map that represents the relevance of each area for memory (Fig. 1*A*,*B*). This approach assumes that genes involved in memory should be highly expressed in the brain areas highly relevant for memory. For instance, this relationship was observed in the case of reward-associated gene *DRD2* in reward processing areas (Mengod et al., 1992; Pappata et al., 2002; Schott et al., 2008). For the genetic expression and functional maps, we used the Allen Human Brain Atlas (AHBA) transcriptome and the Neurosynth “memory” neuroimaging map.

10.1523/ENEURO.0283-19.2019.f1-1 Extended Data Figure 1-1 Visualization of the *GRB14* gene expression from the AHBA. Visualized Allen human brain transcriptome atlas on a single donor showing the gene expression *z* score of regions involved in memory and motor function. Created with the planar viewer at http://human.brain-map.org/. Download Extended Data Figure 1-1, TIF file.

10.1523/ENEURO.0283-19.2019.f1-2 Extended Data Figure 1-2 Visualization of Neurosynth memory and motor neuroimaging maps. Visualized Neurosynth maps using MRIcro showing the *z* score intensity of regions involved in memory and motor function. Only positive *z* scores are used. Download Extended Data Figure 1-2, TIF file.

10.1523/ENEURO.0283-19.2019.f1-3 Extended Data Figure 1-3 Cortical and subcortical regions analyzed. Regions used in the cortical and subcortical analyses, as used in Arnatkevic̆iūtė et al. (2019). The ontology follows AHBA naming convention; however, the hippocampus has been relabeled a subcortical structure in line with neuroimaging convention. Download Extended Data Figure 1-3, XLSX file.

We then identified memory-related genetic profiles in an unsupervised manner with gene set enrichment analysis (GSEA; Fig. 1*C*). Due to the correlational nature of the spatial correlation analysis, we drew on biological knowledge databases (i.e., enrichment analyses with the Gene Ontology library) to guide our identification of biological processes and genes associated with memory. To identify cortex-specific and subcortex-specific memory-associated genes, we compared the differences between their respective gene profiles (Fig. 1*D*). As there are genes that are involved in general memory across cortical and subcortical regions (Gallo et al., 2018), we also characterized the overlap between cortical-subcortical genetic profiles (Fig. 1*D*). Furthermore, to identify candidate genes, we identified the top-10 genes most likely associated with memory with leading-edge analysis (LEA; Fig. 1*E*). We then validated our results by verifying that the genetic profiles corroborate with experimental literature. Finally, we assessed whether our approach was effective and precise (Fig. 1*F*,*G*).

Because of their common and critical involvement in general memory, we analyzed both cortical and subcortical areas involved in memory. Of note, previous studies on functional networks mainly focused on cortical or subcortical analyses due to disparate expression profiles. This may be because of marked differences in neuronal composition, number of layers, and connectivity (O’Leary and Koester, 1993; Yushkevich et al., 2009; Modha and Singh, 2010; Kim et al., 2015). At the genetic level, these differences are mirrored by distinct patterns of both gene expression intensity and variability (Hawrylycz et al., 2012; Richiardi et al., 2015; Fox et al., 2014). Thus, we analyzed their spatial correlations separately, as combining both in the spatial correlation analysis would capture the gross cortico-subcortical differences in expression intensity instead of meaningful inter-regional differences in genetic expression.

Despite a common involvement in general memory, we found largely distinct memory-related biological processes and genes across cortical and subcortical regions. Cortical processes included immune and epigenetic regulation; subcortical processes included neurogenesis and glial cell differentiation. Genes shared across cortical-subcortical regions were involved in the regulation of transcription, synaptic plasticity and glutamate receptor signaling. We show that our approach identified a greater number of memory genes in the memory analysis than expected by chance, and more memory genes than motor function genes. These results provide a better understanding of genetics associated with human memory, and nominate candidate genes for future experimental investigations.

## Materials and Methods

### AHBA transcriptome

The AHBA transcriptome was generated from the normalized mRNA microarray sampling of a combined 3702 sampling sites across six donor brains (Hawrylycz et al., 2012; *N* = 6 left hemispheres, *N* = 2 right hemispheres; Fig. 1*A*; see Extended Data Fig. 1-1 for an example visualization of a gene). The donors were three white males, two African-American males and one Hispanic female. Donor age ranged from 24 to 57, mean donor age was 42.5 years (SD = 11.2 years), Data from all six donors was horizontally concatenated into a .csv file, with one probe per row. For more details on the dataset and data collection procedures, see http://help.brain-map.org/display/humanbrain/Documentation.

### Neurosynth memory association map

Neurosynth ‘memory’ and ‘motor’ association maps [Montreal Neurologic Institute 152 (MNI152) space, thresholded FDR < 0.01] were used as neuroimaging data for the memory and motor functions (Fig. 1*A*; see Extended Data Fig. 1-2 for a visualization of the Neurosynth memory and motor maps). These cognitive functions were chosen as they were largely functionally and anatomically distinct and were constructed from a similar number of studies (N_memory_ = 2744, N_motor_ = 2565). Neurosynth quantifies the relevance of each voxel to the user-specified search terms (e.g., memory) based on a database of neuroimaging studies. In the example of the memory map, each voxel is assigned a *z* score that reflects the preferential association of that voxel with memory, instead of other functions. For instance, the large positive *z* score in the hippocampus means that studies whose abstracts include the word memory are more likely to report hippocampus engagement than studies that do not include the word memory (Yarkoni et al., 2011). Negative *z* scores indicate a higher correlation with other search terms unrelated to memory, and thus were excluded from our analyses. For broad cognitive function domains, single terms enable the generation of maps that approximate the target cognitive process reasonably well (Yarkoni et al., 2011). Therefore, we used memory and motor as our search terms to derive the memory and motor association maps. Note that in the example of memory, this approach resulted in inclusion of a broad range of subfunctions, such as working memory and long-term memory. This allowed for a broader and more inclusive definition of memory and motor function for the subsequent identification of their genetic signatures. In the generation of such maps, it is possible that the foci identified by the automatic coordinate extraction process of Neurosynth may be inaccurate due to different data formats of online neuroimaging journals and sites. However, when compared with the gold standard of manually curated activation foci in the Surface Management Systems Database (SumsDB), automatically extracted coordinates shows high sensitivity (84%) and specificity (97%; Yarkoni et al., 2011). As such, the memory and motor neuroimaging maps used are reasonable approximations of regions involved in general memory and motor function.

### Preprocessing of transcriptome

We followed preprocessing steps as outlined in Arnatkevic̆iūtė et al. (2019), including the brain atlas used to delineate cortical and subcortical regions. Note that this atlas from Arnatkevic̆iūtė et al. (2019) differs from the modified Brodmann atlas used by AHBA, and the hippocampus was relabeled as a subcortical instead of a cortical region in line with human neuroimaging conventions (Hawrylycz et al., 2012; Ji et al., 2019; list of cortical and subcortical areas in Extended Data Fig. 1-3). In preprocessing, probes were first reannotated with the Reannotator package. We excluded the probes which had <50% samples exceeding the background expression level. For each gene, we selected the probe with the highest differential stability score, i.e., with the least spatial variability across donors. The AHBA data were normalized (*z* score) for each donor. Cortical and subcortical regions were normalized separately. This was done to account for individual and cortico-subcortical differences in gene expression (Hawrylycz et al., 2011). This returned a 15,625 gene-by-1285 brain sample matrix for the left cortex, and 15,625-by-497 matrix for the left subcortex, respectively. This gave an individual average of 214 left cortical (range: 175–259) and 83 left subcortical samples per donor (range: 59–115). In the subsequent step below, this was further restricted to the brain regions of interest in co-registration. With the usage of a different brain atlas from AHBA, it is possible that our re-annotation of regions as cortical or subcortical areas may be inaccurate and may affect cortical and subcortical analyses. However, as we retained the AHBA ontological labels and simply re-annotated the hippocampus as subcortex, this step is reasonable.

### Co-registration of AHBA and Neurosynth memory map

To allow a comparison of spatial similarity between neuroimaging and AHBA maps of differing resolutions, both maps were co-registered into a common 3D stereotactic brain space (Fox et al., 2014). This was done by using the MNI coordinates provided by AHBA for representing the transcriptome sampling points in MNI152 template space. This was also the space used by the Neurosynth map. The Neurosynth map was used as a mask for the AHBA map, so that only the overlapping areas were included in the correlation analysis (Fox et al., 2014). Due to the limited availability of hemispheres sampled (six left and two right hemispheres), we used only the left hemispheres, separated into cortical and subcortical regions. In subsequent steps, the cortical and subcortical analyses were kept separate. Besides providing insight into the separate cortical and subcortical genetic mechanisms, this also avoided confounds from their divergent transcriptional profiles (Richiardi et al., 2015). We then matched the smoothing of both maps by smoothing the AHBA with a 6mm radius sphere. At the end of this step, there remained on average 93 memory (range: 72–107) and 65 motor cortical (range: 55–76) data points, and on average 40 memory (range: 25–71) and 43 motor subcortical (range: 24–69) data points per individual. It is possible that the coregistration of AHBA and Neurosynth maps may be affected by errors introduced during the MRI to MNI coordinate transformation by AHBA. However, the Allen Institute transformed the MRI to MNI coordinates using standard methods for *in cranio* and *ex cranio* brains (four donor brains were imaged *ex cranio*), and we ensured reasonable coregistration by visually inspecting the resulting maps.

### Spatial correlation analysis of AHBA and Neurosynth data

To obtain spatial correlation values per gene, we relied on a tool that correlates the spatial AHBA and neuroimaging maps (Yarkoni et al., 2011; Fox et al., 2014). Each datapoint used in correlation is a point in space, with a normalized gene expression intensity value and a neuroimaging map *z* score. For a gene associated with memory, we would expect high spatial similarity between both AHBA and Neurosynth maps, i.e., a pattern of high gene expression within areas highly relevant for memory and vice versa. This would be reflected in a high mean correlation value for that gene. We applied the spatial analysis separately for cortical and subcortical regions (Fig. 1*B*). An approximate random effects analysis was used to account for individual gene expression variability and to counter the sparse cortical sampling in the AHBA maps. Donor regression slope and intercept were modeled individually. We subsequently obtain each gene’s mean correlation value (averaged across the six donors), which was the statistic of interest. From this step, we obtained four lists *L* of 15,625 genes, for memory and motor function, and the respective cortical and subcortical regions.

### Identifying biological processes of cortical and subcortical memory

We used a gene set analysis tool (GSEA Pre-ranked, GenePattern module, version 6.0.5) to identify sets of genes associated with common biological functions (Fig. 1*C*). The four lists of genes *L* were ranked by mean correlation value (the ranking statistic used in this case) and passed to GSEA Pre-ranked. We analyzed each list *L* with GSEA Pre-ranked with the default parameters, including weighted scoring using the Gene Ontology Biological Process library (c5.bp.v6.0.symbols.gmt). GSEA Pre-ranked looks separately at the top and bottom of each list *L* for genes that overlap with each gene set in the database (Mootha et al., 2003; Subramanian et al., 2005). This overlap or gene set enrichment was assessed by weighted scoring based on mean correlation (*r* value). This returns a normalized enrichment score, a significance *p* value, and an FDR *q* value (across all gene sets tested) for each enriched gene set. From the top positively and negatively correlated genes in each list *L*, we obtained separate sets *S^+^* and *S^−^* of positively and negatively enriched gene sets, respectively. For subsequent analyses, we only used all gene sets with FDR *q* < 0.05. Note that the motor cortical (–) analysis (negatively correlated genes from the motor cortical analysis) did not have any gene sets surviving FDR < 0.05, and thus was not used in subsequent analyses, i.e., biological processes nor candidate genes as output. Thus, this effectively meant eight *S^+^* and seven *S^−^* usable sets for subsequent steps. For this analysis and subsequent steps, genes that are found in ≥1 significantly enriched gene set are termed memory genes, as opposed to the top-10 memory genes identified below.

### Visualization of significantly enriched gene sets

To identify the overall biological themes across gene sets. we grouped gene sets into networks by the genes that they share (Fig. 1*C*). For each pair of sets *S^+^* and *S^−^*, we input their gene sets into the Cytoscape network visualization software, and included the gene sets with FDR *q* < 0.05. We then used the Enrichment Map app to construct the gene set networks and annotated them with the Wordcloud extension for subsequent interpretation (Cline et al., 2007; Merico et al., 2010; Oesper et al., 2011). This was done using the default settings except for a custom FDR *q* value threshold of 0.05 (i.e., FDR < 0.05). This step returned four annotated enrichment maps for the list *L* of each cognitive function and for each of cortical and subcortical areas.

### Functional annotation of overlapping genes

We used the ToppGene suite (with Gene Ontology Biological Process library) to functionally cluster memory genes (identified in the GSEA analysis) that are (1) cortex specific, (2) subcortex specific, (3) and shared between both (Fig. 1*D*; Chen et al., 2009). From the output, we thresholded biological process gene sets as those that satisfied FDR < 0.05. This returned three lists of gene sets, one for each type of gene above.

### Identifying candidate genes associated with cortical and subcortical memory

To identify the top-10 genes most likely to be relevant to the cognitive function, we identified genes frequently appearing across the gene sets with the LEA (Fig. 1*E*; Mootha et al., 2003; Subramanian et al., 2005). For the analysis of each cognitive function in cortical and subcortical regions, we input the respective gene sets with FDR *q* < 0.05 (javaGSEA desktop application). LEA then identified the genes that appeared frequently across the leading-edge subset genes across gene sets in *S^+^* or *S^−^* (Subramanian et al., 2005; Fleming and Miller, 2016). We ranked genes by the number of leading-edge gene sets they enrich; in the case of a tie in the number of gene sets, we rank them by the mean spatial correlation value. The top-10 genes appearing most frequently in the positively and negatively enriched gene sets were designated as the candidate gene list *CL*. The outputs were seven candidate gene-cognition association lists *CL* of 10 genes each for all *S^+^* and *S^−^*.

### Literature review of genetic signatures

To quantify the number of candidate gene “hits” for the memory analysis, we conducted a literature review for each gene list *CL* and counted the number of gene-memory (i.e., true positives) or gene-motor function associations (i.e., false positives; Fig. 1*F*). This was done by reviewing experimental literature on Google Scholar, via a search query: [“gene name” AND (“memory” OR “amnesia” OR “Alzheimer’s” OR “dementia”)] and [“gene name” AND (“motor function” OR “motor coordination” OR “locomotor” OR “ataxia” OR “motor learning” OR “Parkinson’s” OR “Huntington’s”)], respectively. The same was repeated for the motor analysis for the respective true positives and false positives. The disorders were selected for keyword search because they prominently feature deficiencies in memory and motor functioning. Strong evidence included studies that employed *in vivo* gene manipulations, mutants and pharmacological interventions, while weak evidence included computational gene associations, *in vitro* studies, differential gene expression studies and human case studies. Literature evidence only counted as validation if it implicated the corresponding brain area, i.e., cortical or subcortical. As such, evidence of a given gene’s role solely in the non-analyzed brain region was not counted. For example, if a paper showed that the knock-out of gene A solely in the subcortex leads to memory deficits, it would not count as evidence for the analysis of cortical memory.

### Correlation difference in memory and motor analyses

If the method is valid, memory genes should have a higher average correlation value from the memory analysis compared with the motor analysis, and vice versa for motor genes and the motor analysis *r* value. For each gene, this was calculated by subtracting its motor function *r* value from the memory *r* value, with a positive difference counting toward the method’s effectiveness (Fig. 1*G*). Note that for the memory *r* values from the negative gene lists (e.g., memory cortical -), we multiply the *r* value difference with –1 to express this difference as a positive value, consistent with the positive memory gene lists. We then take the average of all genes for each set *S* that satisfy FDR *q* < 0.05 (same threshold as enrichment map visualization) to obtain seven such values. As the number of genes per set *S* is different, we bootstrapped the number of correlation difference values used for calculating the average correlation difference value per set. This was done separately for the memory and motor analyses by repeatedly subsampling the correlation differences (10,000 iterations) to the minimum number of genes in memory (*n* = 231) and motor sets (*n* = 146), respectively. We visualized this as a boxplot for each of the seven sets, with the bootstrapped mean and 95th percentiles (whiskers) for memory and motor analyses. If the baseline does not fall within the 95th percentile distribution (i.e., whiskers do not overlap with the baseline of zero) the score is deemed significantly different from baseline (*p* < 0.05).

### Assessing method effectiveness in identifying candidate genes

We quantified method effectiveness based on the prior literature review (Fig. 1*G*). To do so, we calculated the chance probability of obtaining *N* memory genes per gene list. This is done by selecting *N* memory genes (without replacement) from the pool of known memory or memory-related disorder genes (*n* = 644) out of all 15,625 genes analyzed. For example, if 10 out of 10 genes in the gene list are memory genes, the chance probability of this occurring is 1.32 × 10^−14^. The same was done accordingly for motor function and the motor function genes (*n* = 104). These memory genes were compiled from three sources: (1) the literature review above; (2) the biological function gene sets “GO:0007611 Learning or memory,” from database AmiGO_2_ (Carbon et al., 2009; version 2.4.26, release date 2016-08); and (3) van Cauwenberghe et al. (2016). The motor-related genes (motor or motor-related disorder) were obtained from (1) the literature review above, (2) the biological function gene sets “GO:0061743 motor learning” and “GO:0061744 motor behavior” from database AmiGO_2_ (Carbon et al., 2009), and (3) Lin and Farrer (2014).

### Precision score for memory and motor analyses

We asked, of a given memory gene list with genes labeled as memory genes, how many of these are actually related to memory. We quantified this by calculating a precision score (Fig. 1*G*). We first determined the true positives (i.e., genes associated with memory from the literature review) and false positives (i.e., genes associated with motor function). The literature evidence was weighted such that for true positives, strong evidence and weak evidence (defined above) received a full point and half-point respectively. For each gene list, we then determined the method’s precision score by dividing “true positives” by the sum of true positives and false positives (Eqs. 1, 2). If the method is precise, for memory analyses, the memory precision scores should be above 0.5 and motor score below 0.5, and vice versa. We plotted the memory and motor precision scores for each gene list (ranging from 0 to 1), and the difference between these scores (ranging from –1 to 1). Ideally, the difference should be greater than zero. In the following equations,

(1) $$\text{Memory precision score}=\frac{\left(0.5\;{Memory}_{w}+{Memory}_{s}\right)}{\left((0.5\;{Memory}_{w}+{Memory}_{s})+(0.5\;{Motor}_{w}+{Motor}_{s})\right)},$$

(2) $$\text{Motor precision score}=\frac{\left(0.5\;{Motor}_{w}+{Motor}_{s}\right)}{\left((0.5\;{Memory}_{w}+{Memory}_{s})+(0.5\;{Motor}_{w}+{Motor}_{s})\right)},$$

*Memory_s_* = number of genes with strong evidence for its association with memory; *Memory_w_* = number of genes with weak evidence for its association with memory; *Motor_s_* = number of genes with strong evidence for its association with motor function; and *Motor_w_* = number of genes with weak evidence for its association with motor function.

### Data availability statement

All genetic and neuroimaging data used are available from the AHBA (https://human.brain-map.org) and Neurosynth (https://www.neurosynth.org). The scripts for preprocessing the transcriptome are available at https://github.com/BMHLab/AHBAprocessing. The correlation scripts and input data are available for non-commercial use in Extended Data 1 and at https://github.com/PK-HQ/geneCognitionDiscovery.

## Results

### AHBA and Neurosynth maps

For identifying whole-brain adult human memory genes, we first needed to conduct the spatial correlation analysis between 3D high-resolution neuroimaging and transcriptome maps of the adult human brain. As such, we used the high density, whole human brain AHBA transcriptome, and the Neurosynth memory association map of each voxel’s association with memory in general as input datasets (Yarkoni et al., 2011).

The AHBA was derived from six donor brains, and contains whole genome human brain gene expression in the left cortical and subcortical regions (*N* = 6; Fig. 1*A*; see example visualization in Extended Data Fig. 1-1; Hawrylycz et al., 2012). The Neurosynth memory association map is a meta-study map (*N* = 2744) which represents each brain voxel’s relevance for memory (as opposed to other cognitive functions), specified by positive *z* scores (Fig. 1*A*; see visualization of memory and motor function maps in Extended Data Fig. 1-2; Yarkoni et al., 2011). Note that the usage of memory here refers to memory in general, as the map was constructed from memory-related neuroimaging studies that employ multiple types of memory tasks (Yarkoni et al., 2011). We co-registered both maps into a common MNI152 space. The memory areas in the memory association map were used to define the usable AHBA samples for the subsequent spatial correlation analysis.

### Spatial similarity analysis

**Figure 2.**
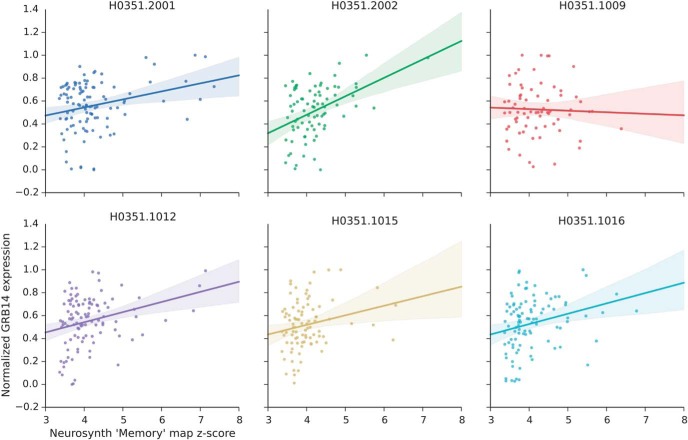
An example of spatial similarity analysis output. The expression levels of the top-correlated cortical gene, *GRB14*, is visualized as a function of the Neurosynth map’s voxel-wise relevance to memory function (*z* score). Normalized gene expression (*y*-axis) plotted against neuroimaging map *z* scores (*x*-axis). Each colored regression line represents the best-fit line for each of six donors (colors); the translucent band around each line represents the 95% confidence interval estimate.

Using these datasets, we sought to isolate the genes with high spatial correlation values between their gene expression and memory term maps for subsequent analysis steps, as they are most likely related to memory (Fox et al., 2014). We conducted the spatial similarity analysis between the AHBA and Neurosynth association maps separately for cortical and subcortical regions due to their marked differences (see Introduction; the list of cortical and subcortical regions is available in Extended Data Fig. 1-3), and for memory and motor function (see an example of spatial correlation in Fig. 2). Each analysis yielded a list *L*, which contained the mean correlation values of 15,625 genes used for subsequent ranking (Fig. 1*B*).

We subsequently ranked each list *L*. A positive correlation indicates higher gene expression in areas relevant for memory, and a negative correlation implies lower expression in areas relevant for memory. The top-10 positively and negatively correlated genes for the memory cortical and subcortical analyses are shown in Table 1 (see the spatial correlation value of all genes in Extended Data Table 1-1). There were more negatively correlated genes than positively correlated genes for both cortical and subcortical analyses of memory (Extended Data Table 1-1). We found 8383 positively and 7243 negatively correlated genes for the cortical areas, and 7642 positively and 7984 negatively genes for the subcortical areas.

**Table 1.** Spatial correlation analysis for memory function

Correlation polarity	Cortical analysis			Subcortical analysis	
	Gene	Mean *r*		Gene	Mean *r*
+	*GRB14*	0.24		*NEUROD6*	0.66
	*DYRK3*	0.21		*NEUROD1*	0.66
	*FILIP1*	0.21		*NPTXR*	0.65
	*SPHKAP*	0.21		*PLEKHG5*	0.65
	*TMTC1*	0.21		*NNMT*	0.64
	*TSPAN2*	0.21		*LRRC2*	0.64
	*S100A10*	0.21		*C9orf16*	0.64
	*HEYL*	0.20		*MICAL2*	0.64
	*FZD7*	0.20		*SLC17A7*	0.64
	*KCTD12*	0.20		*DUSP4*	0.64
−	*NRAP*	–0.25		*CRNDE*	–0.64
	*DLGAP1-AS4*	–0.22		*FAM222A*	–0.63
	*CTNNAL1*	–0.21		*CRABP1*	–0.62
	*FGF18*	–0.20		*NTM-AS1*	–0.62
	*MIR124-2HG*	–0.19		*SELENOP*	–0.62
	*HIST1H1D*	–0.19		*KIF19*	–0.61
	*TDRD1*	–0.19		*LOC100506725*	–0.61
	*SLC24A4*	–0.18		*CA14*	–0.61
	*CCDC144B*	–0.18		*ZFHX4*	–0.61
	*LINC00476*	–0.18		*LINC00844*	–0.61

Top-10 positively and negatively correlated genes from the memory analysis, ranked by the mean correlation magnitude across six donor brains. The positively and negatively correlated genes are listed separately for cortical and subcortical areas. See Extended Data Table 1-1 for the complete list of genes and respective *r* values.

10.1523/ENEURO.0283-19.2019.t1-1 Extended Data Table 1-1 Spatial similarity values for 15,625 genes. Spatial correlation values for all genes used in the memory and motor spatial similarity analysis. Download Table 1-1, XLSX file.

10.1523/ENEURO.0283-19.2019.ed1 Extended Data 1 Code, input and output data for the correlation analysis, containing preprocessed data for input into the spatial similarity analysis script, and output data for usage with GSEA Pre-ranked. Download Extended Data 1, ZIP file.

### Distinct gene expression profiles associated with cortical and subcortical memory

Following the spatial correlation analyses, we aimed to define the gene expression profiles related to cortical and subcortical memory in a comprehensive manner. To identify and characterize sets of genes that work toward a common biological function (i.e., gene sets), we analyzed each of the cortical and subcortical lists *L* with GSEA Pre-ranked (Fig. 1*C*). This yielded positively scoring and negatively scoring gene sets, derived from the positively and negatively correlated genes of *L*, respectively. These gene sets were then grouped into functionally related clusters, and automatically annotated with biological themes (Cline et al., 2007; Merico et al., 2010; Oesper et al., 2011).

**Figure 3.**
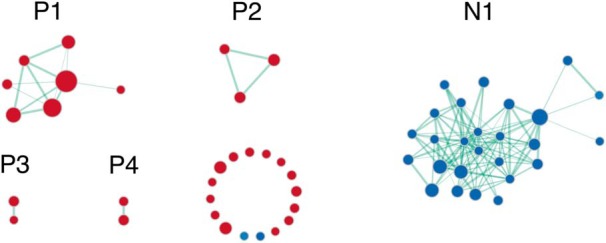
Enrichment map visualization for cortical memory. Clusters are labeled with P for positive, N for negative. Gene set clusters were found to be related to memory. Positive clusters were associated with immune signaling, calcium transport and actin filament assembly. The negative cluster contained gene sets involved in chromatin dynamics and epigenetic regulation. See Extended Data Figure 3-1 for the full output from GSEA Pre-ranked.

Overall, the cortex and subcortex had distinct biological themes that were previously found associated with memory. For cortical memory, GSEA revealed 28 positive and 29 negative significantly enriched gene sets. Visualization of the enrichment network showed that these gene sets were grouped into five distinct clusters (Fig. 3; the complete GSEA results are in Extended Data Fig. 3-1), with gene sets within each cluster sharing enriched genes. These gene sets were found to be related to memory. The positive cluster P1 contained gene sets implicated in immune response and Fcγ receptor signaling (Fernandez-Vizarra et al., 2012; Marin and Kipnis, 2013). P2 was implicated in interferon gamma signaling (Litteljohn et al., 2014), P3 in transmembrane calcium ion transport and P4 in actin filament assembly (Krucker et al., 2000; Lamprecht, 2011). The negative cluster N2 contained gene sets involved in chromatin dynamics, epigenetic regulation, and immune cell differentiation (Kim and Kaang, 2017).

10.1523/ENEURO.0283-19.2019.f3-1 Extended Data Figure 3-1 GSEA output for the memory cortical analysis, containing all gene sets tested and their respective enrichment scores, *p* values, and FDR values for the memory cortical analysis in GSEA Pre-ranked. Download Figure 3-1, XLS file.

**Figure 4.**
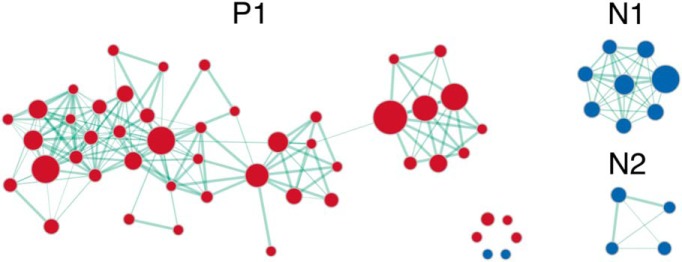
Enrichment map visualization for subcortical memory. Clusters are labeled with P for positive, N for negative. Gene set clusters were found to be associated with memory. Positive clusters were associated with synaptic transmission, long-term plasticity, glutamate signaling, and neurite morphogenesis. Negative clusters included gene sets involved in transcription and translation, and glial cell differentiation. See Extended Data Figure 4-1 for the full output from GSEA Pre-ranked.

For subcortical memory, GSEA revealed 50 positive and 14 negative significantly enriched gene sets. Visualization of the enrichment network showed that these gene sets were grouped into three distinct clusters (Fig. 4; the complete GSEA results are in Extended Data Fig. 4-1). Similarly, these gene sets were found to be related to memory. The positive cluster P1 is implicated in synaptic transmission and synaptic plasticity. It also included gene sets involved in endocytosis and exocytosis, neurotransmitter secretion, long-term potentiation (Stuchlik, 2014), glutamate receptor signaling, and neuron projection morphogenesis (Kasai et al., 2010). The negative cluster N1 is related to transcription and translation processes (Jarome and Helmstetter, 2014; Alberini and Kandel, 2015), and cluster N2 to glial cell and oligodendrocyte differentiation (Hertz and Chen, 2016; Pepper et al., 2018).

10.1523/ENEURO.0283-19.2019.f4-1 Extended Data Figure 4-1 GSEA output for the memory subcortical analysis, containing all gene sets tested and their respective enrichment scores, *p* values, and FDR values for the memory subcortical analysis in GSEA Pre-ranked. The motor cortical and subcortical analyses are also included here. Download Figure 4-1, XLS file.

**Figure 5.**
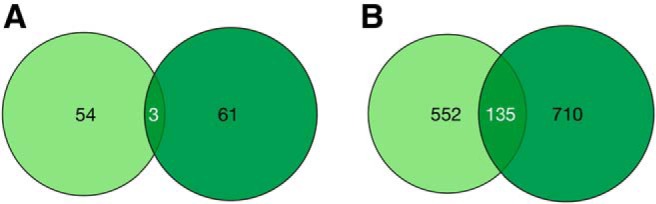
Overlap between cortical and subcortical memory gene sets and genes. ***A***, Number of overlapping cortical and subcortical memory gene sets derived from GSEA. ***B***, Number of overlapping cortical and subcortical memory genes derived from GSEA. Light green denotes cortical genes, dark green denotes subcortical genes. See Extended Data Figure 5-1 for the list of gene sets and genes that are shared or distinct across cortex and subcortex.

To identify differences and overlaps in the cortical and subcortical genetic profiles, we identified and characterized the different and shared (1) biological processes as shown in the enrichment maps, and (2) memory genes (i.e., all genes found in ≥1 enriched gene set; Fig. 1*D*). We found a low overlap of 2.5% of gene sets (*N* = 3) and 9.6% of genes (*N* = 135) between cortical and subcortical regions (Fig. 5; the complete list of distinct and overlapping genes is in Extended Data Fig. 5-1). The overlapping genes were involved in memory-related processes of protein transport, transcriptional regulation, synaptic plasticity and glutamate receptor signaling (Peng et al., 2011; Rosenberg et al., 2014; Alberini and Kandel, 2015; Table 2; full output of gene sets and genes from ToppGene in Extended Data Table 2-1). These include genes involved in the Arp2/3 complex, GABA and AMPA ligand-gated ion channels which are critical for memory function (Gasbarri and Pompili, 2014; Basu et al., 2016; Takemoto et al., 2017; Extended Data Table 2-1). Cortex-specific genes were involved in memory-associated processes such as DNA repair, epigenetic regulation, immunity and IFN-γ signaling (Marin and Kipnis, 2013; Litteljohn et al., 2014; Kim and Kaang, 2017; Hou et al., 2018; Extended Data Table 2-1). Subcortex-specific genes are involved in neurogenesis, dendrite morphogenesis, glial cell differentiation and myelination (Hertz and Chen, 2016; Kao et al., 2018; Pepper et al., 2018; Extended Data Table 2-1). Note that the same gene set can appear both in the cortical-specific and subcortical-specific biological processes. For instance, the memory gene sets is enriched in both regions, but in each case, the gene set enrichment is driven by distinct genes (Extended Data Table 2-1). This is because different genes can be relevant for, and thus increase enrichment for the same biological process gene set.

**Table 2.** Comparison of cortical and subcortical genes associated with memory

	GO term	*p*	*q*
Common across cortex and subcortex	Protein targeting to ER	8.2 × 10^–67^	1.9 × 10^–63^
	mRNA catabolic process	3.5 × 10^–41^	8.5 × 10^–39^
	Regulation of synaptic plasticity	4.3 × 10^–9^	1.1 × 10^–7^
	Glutamate receptor signaling pathway	4.1 × 10^–9^	1.1 × 10^–7^
Cortex only	DNA repair	6.2 × 10^–29^	7.3 × 10^–27^
	Regulation of gene expression, epigenetic	3.7 × 10^–27^	3.8 × 10^–25^
	Interferon-gamma-mediated signaling pathway	3.4 × 10^–23^	2.4 × 10^–21^
	Regulation of neurogenesis	8.9 × 10^–16^	4.1 × 10^–12^
Subcortex only	Neurogenesis	3.6 × 10^–117^	5.6 × 10^–114^
	Neuron projection morphogenesis	5.4 × 10^–96^	2.4 × 10^–93^
	Glial cell differentiation	6.0 × 10^–42^	7.5 × 10^–40^
	Myelination	2.1 × 10^–30^	1.6 × 10^–28^

We identified biological processes linked to memory genes shared or distinct across cortex and subcortex. GO = gene ontology biological process library; *p* and *q* refer to *p* value and FDR *q* value, respectively. See Extended Data Table 2-1 for the complete list of enriched gene sets and genes from ToppGene.

10.1523/ENEURO.0283-19.2019.t2-1 Extended Data Table 2-1 ToppGene output for the distinct and overlapping genes between cortex and subcortex. Enriched gene sets of the cortex-specific genes, subcortex-specific genes, and overlapping genes, including genes in the gene sets. Download Table 2-1, XLSX file.

10.1523/ENEURO.0283-19.2019.f5-1 Extended Data Figure 5-1 List of distinct and overlapping memory genes between cortex and subcortex. All memory genes derived from the prior GSEA step that are cortex specific, subcortex specific, or shared between cortex and subcortex. It includes motor genes as well. Download Figure 5-1, XLSX file.

### Core differentially expressed genes related to cortical and subcortical memory

To identify the top-10 memory genes that are most likely linked to human memory function for future experimental investigation, we identified genes relevant for multiple gene sets obtained above with the LEA (Fig. 1*E*; Subramanian et al., 2005; Darby et al., 2016; Fleming and Miller, 2016). Previous work has shown that such genes that drive the enrichment of multiple gene sets are more likely related to the phenotype analyzed, i.e., memory function in this case (Subramanian et al., 2005; Darby et al., 2016; Fleming and Miller, 2016). The combination of GSEA and LEA were previously effective in identifying genetic signatures of cognitive functions (Thomassen et al., 2008; Ersland et al., 2012; Lee et al., 2013), including episodic and working memory (Heck et al., 2014; Luksys et al., 2015). We applied LEA to the positively and negatively scoring gene sets above, followed by selecting the top-10 genes appearing most frequently across the leading-edge subsets of the gene sets. These genes were then validated with animal model literature, which were classified as strong or weak evidence supporting the link between the gene and memory function (Fig. 1*F*). Strong evidence was comprised of gene manipulation or drug treatment studies, e.g., gene knock-out leading to memory alteration. Weak evidence encompassed correlational or computational studies, such as gene upregulation that correlated with enhanced memory performance.

For cortical memory, nine out of 10 positively correlated genes were previously implicated in memory function (Table 3**;** full list of cortical memory genes and literature review Extended Data Table 3-1, complete LEA output in Extended Data Table 3-2). Genes *PRKCD* (Etcheberrigaray et al., 2004; Conboy et al., 2009), *RAC1* (Haditsch et al., 2009; Oh et al., 2010), *LIMK1* (Todorovski et al., 2015), and *CDC42* (Kim et al., 2014; Zhang et al., 2016) had strong associations with memory. For the corresponding negatively correlated candidate genes, all 10 genes had strong evidence supporting their role in memory. These were all genes encoding the histone H4 protein, which was linked to memory performance (Peleg et al., 2010). Deregulation of histone H4 acetylation in aged mice was linked to memory impairment, and reinstating this regulation improved their memory.

**Table 3.** Candidate gene lists from cortical analyses of memory

CL	Gene	# leading edge subsets	mean *r*	Associated cognitive function			
				Mem_s_	Mem_w_	Mot_s_	Mot_w_
Memory Cortical +	*PRKCD*	9	0.10	Y			
	*RAC1*	9	0.07	Y			
	*HLA-DRB5*	8	0.12		Y		
	*HLA-DRB1*	8	0.09		Y		
	*HLA-DRB4*	8	0.09		Y		
	*HCK*	8	0.09		Y		
	*HLA-DRB3*	8	0.08				
	*LIMK1*	7	0.13	Y			Y
	*CDC42*	7	0.11	Y			
	*VAV1*	6	0.15		Y		
Memory Cortical −	*HIST1H4A*	23	–0.18	Y			Y
	*HIST1H4E*	23	–0.17	Y			Y
	*HIST1H4H*	23	–0.13	Y			Y
	*HIST1H4I*	23	–0.12	Y			Y
	*HIST4H4*	23	–0.11	Y			Y
	*HIST1H4J*	23	–0.11	Y			Y
	*HIST1H4B*	23	–0.10	Y			Y
	*HIST1H4K*	23	–0.10	Y			Y
	*HIST1H4F*	23	–0.09	Y			Y
	*HIST1H4D*	23	–0.09	Y			Y

Candidate gene lists for the memory analysis of cortical regions, from positively and negatively correlated gene lists. Genes are ranked by the number of leading-edge subsets they appear in, and subsequently by mean *r* value. *CL*: candidate gene list; # leading-edge subsets: number of leading-edge subsets that the gene was found in; Mem_s_: strong evidence for memory function; Mem_w_: weak evidence for memory function; Mot_s_: strong evidence for motor function; Mot_w_: weak evidence for motor function; +: positively correlated candidate gene list; −: negatively correlated candidate gene list. See Extended Data Table 3-1 for the literature review supporting the cortical gene-cognition associations and Extended Data Table 3-2 for the complete LEA output for cortical analyses.

10.1523/ENEURO.0283-19.2019.t3-1 Extended Data Table 3-1 Candidate genes for the cortical memory analysis. Literature review for the candidate genes derived from the LEA of cortical memory gene sets, ranked by the number of gene sets it is found in. Download Table 3-1, XLSX file.

10.1523/ENEURO.0283-19.2019.t3-2 Extended Data Table 3-2 LEA output for the memory cortical analysis, containing genes and the significant gene sets it is shared across (FDR < 0.05) from the LEA of memory cortical analysis gene sets. Download Table 3-2, XLSX file.

For subcortical memory, all 10 positively correlated genes were previously implicated in memory function (Table 4; full list of subcortical memory genes and literature review in Extended Data Table 4-1; LEA results in Extended Data Table 4-2). Genes *CDK5*, *NLGN1*, *RAB3A*, *STX1A*, *SNCA*, *SYT1*, and *UNC13A* were strongly linked to memory (Fujiwara et al., 2006; Yang et al., 2007; Liu et al., 2009; Guan et al., 2011; Kokhan et al., 2012; Bie et al., 2014; Mishiba et al., 2014; Böhme et al., 2019). Seven out of 10 negatively correlated candidate genes had weak evidence implicating them in memory. These were genes encoding ribosomal subunits, which were differentially expressed in rodents that display better memory performance (Wang et al., 2003; Kong et al., 2009; Winbush et al., 2012; Katz and Lamprecht, 2015; Oka et al., 2016; Zhang et al., 2018).

**Table 4.** Candidate gene lists from subcortical analyses of memory

CL	Gene	# leading edge subsets	mean *r*	Associated cognitive function			
				Mem_s_	Mem_w_	Mot_s_	Mot_w_
Memory subcortical +	*CDK5*	27	0.26	Y			Y
	*NLGN1*	26	0.51	Y			
	*UNC13B*	26	0.38		Y		
	*RAB3A*	25	0.40	Y		Y	
	*STX1A*	24	0.57	Y			
	*SYT12*	23	0.44		Y		
	*STX1B*	22	0.45		Y		
	*SNCA*	21	0.44	Y		Y	
	*SYT1*	21	0.39	Y			Y
	*UNC13A*	20	0.46	Y			Y
Memory subcortical –	*RPL34*	8	–0.54		Y		
	*RPS12*	8	–0.49				
	*RPS13*	8	–0.47		Y		
	*RPS15A*	8	–0.44		Y		
	*RPS29*	8	–0.44				
	*RPL11*	8	–0.44		Y		
	*RPL37A*	8	–0.44				
	*RPL10*	8	–0.44		Y		
	*RPS25*	8	–0.44		Y		
	*RPS27*	8	–0.43		Y		

See Table 2 for notation, Extended Data Table 4-1 for the literature review supporting the subcortical gene-cognition associations, and Extended Data Table 4-2 for the complete LEA output for subcortical analyses.

10.1523/ENEURO.0283-19.2019.t4-1 Extended Data Table 4-1 Candidate genes for the subcortical memory analysis. Literature review for the candidate genes derived from the LEA of subcortical memory gene sets, ranked by the number of gene sets it is found in. Download Table 4-1, XLSX file.

10.1523/ENEURO.0283-19.2019.t4-2 Extended Data Table 4-2 LEA output for the memory subcortical analysis, containing genes and the significant gene sets it is shared across (FDR < 0.05) from the LEA of memory subcortical analysis gene sets. The motor cortical and subcortical analyses are also included here. Download Table 4-2, XLSX file.

### Performance assessment of framework

**Figure 6.**
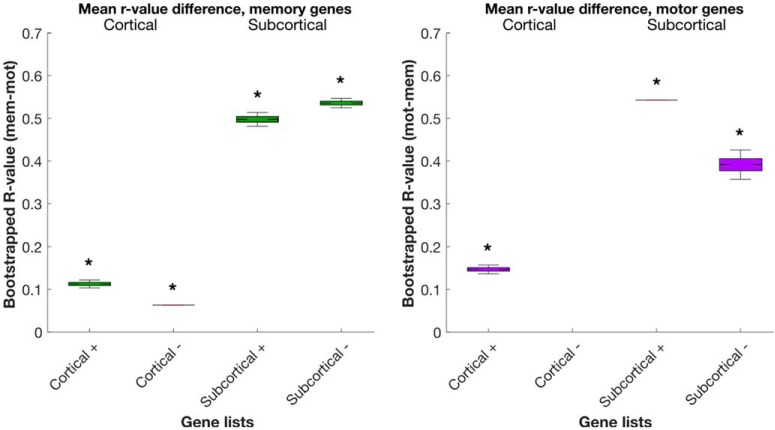
Bootstrapped correlation value differences for all cortical and subcortical candidate genes of memory and motor analysis. For a given memory gene, we calculated the difference between memory and motor analysis *r* values by subtracting motor *r* from memory *r*. If the memory *r* was negative, we took the negative of the difference (to get a positive value). Vice versa for the motor genes. For each cognitive function, we subsampled the number of genes used to the lowest number for calculating the bootstrapped mean difference (231 memory genes and 146 motor genes, respectively, 10,000 iterations). If the 95th percentile did not overlap with the baseline of zero, the bootstrapped difference is considered significant (*p* < 0.05). Note that for the motor cortical analysis, no negatively correlated genes survived the threshold and thus no motor cortical (–) gene list is shown here. See Extended Data Figure 6-1 for the complete list of correlation value differences for genes used in the bootstrap analysis. *denotes *p* < 0.05.

**Figure 7.**
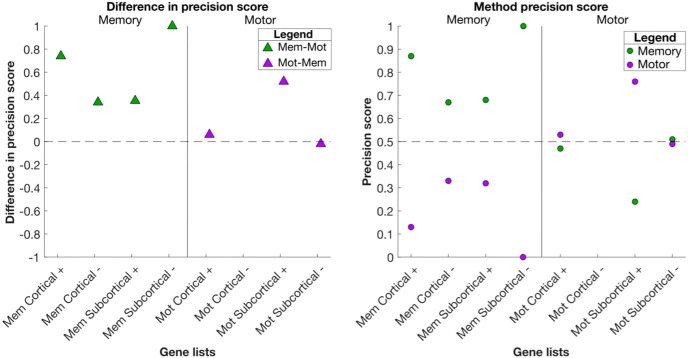
Precision scores for top-10 cortical and subcortical candidate genes of memory and motor analysis. For a given memory gene list, we calculated the memory and motor precision scores with Equations 1, 2 and their difference. Ideally, memory gene lists should obtain a memory score above 0.5, and a motor score below 0.5, and vice versa for the motor genes. Note that for the motor cortical analysis, no negatively correlated genes survived the threshold and thus no motor cortical (–) gene list is shown. See Extended Data Figure 7-1 for the candidate genes of each analysis and the derived method precision score for each gene list.

If our unsupervised approach is valid, for the memory analysis we expect that memory genes should have a higher correlation value from the memory analysis compared with the motor analysis (i.e., sanity check; Figs. 1*G*, 6). Furthermore, in the top-10 memory genes, we expect that a greater number of memory genes in the memory analysis than expected by chance (i.e., statistical significance; Tables 5, 6), and that we find more memory genes than motor function genes (i.e., method precision; Fig. 7).

10.1523/ENEURO.0283-19.2019.f6-1 Extended Data Figure 6-1 Correlation value differences for memory and motor function genes. For each gene derived from the LEA of memory and motor function gene sets (FDR < 0.05), we extracted the memory and motor correlation analysis *r* values and calculated the difference. This was used in the bootstrapped correlation values. Download Figure 6-1, XLSX file.

**Table 5.** Probability of observing the number of memory or motor genes by chance

Cognitive function	Brain region	Gene list	# cognitive function genes (of 10)	*p*
Memory	Cortical	+	9	3.3 × 10^–13^
		–	10	1.3 × 10^–14^
	Subcortical	+	10	1.3 × 10^–14^
		–	7	2.0 × 10^–10^
Motor	Cortical	+	6	7.5 × 10^–14^
		–	n/a	n/a
	Subcortical	+	8	2.9 × 10^–18^
		–	10	1.1 × 10^–22^

We calculated the chance probability of obtaining *N* memory genes per gene list (without replacement), by using the proportion of known memory genes out of the 15,625 genes analyzed. Vice versa for motor genes. Note that for the motor cortical (–) analysis, no genes survived the threshold, and thus, no gene list is shown here. See Extended Data Table 5-1 for the known memory and motor function genes and derived effectiveness scores across all gene lists.

10.1523/ENEURO.0283-19.2019.t5-1 Extended Data Table 5-1 Method effectiveness score for all gene lists, containing known memory and motor function genes, and the probabilities of obtaining *n* memory and *n* motor genes from this pool without replacement, respectively. Download Table 5-1, XLSX file.

**Table 6.** Statistical table

Results section	Data structure	Type of test	Power
Figs. 3, 4	Gene set enrichment analysis	Kolmogorov–Smirnov test	FDR < 0.05
Figs. 5, 6	Distribution of bootstrapped correlation *r* value difference	Overlap of 95th percentiles with baseline of zero	*p* < 0.05
Table 4	Distribution of known memory and motor genes out of 15,625 genes	Probability of obtaining *n* memory/motor genes out of 10 without replacement	*p* < 0.001

Statistical tests for the gene set enrichment analysis, bootstrapped correlation *r* value differences between the memory and motor analyses and the probability of obtaining *n* memory/motor genes by chance from a known pool of memory and motor genes.

10.1523/ENEURO.0283-19.2019.f7-1 Extended Data Figure 7-1 Method precision score for all gene lists. Method precision score calculated for memory and motor gene lists, according to Equations 1, 2. Download Figure 7-1, XLSX file.

Using the candidate gene correlation values, we show that the memory genes displayed a significant positive difference between memory analysis *r* value and motor analysis *r* value, as the 95th percentile (whiskers) did not overlap with zero (Fig. 6; all gene correlation values used in the bootstrap analysis in Extended Data Fig. 6-1). As such, our approach performs as expected.

We found that the method was highly effective. For all memory cortical and subcortical gene lists, the probability of obtaining the number of memory genes observed was significantly above chance (Table 5; full list of memory-related and motor function-related genes that constitute the chance probability in Extended Data Table 5-1). Likewise, for all motor cortical and subcortical gene lists, the probability of deriving the number motor genes observed was highly significant as well.

Using the putative gene functions inferred from the literature review, we also found that the method had high precision, as the difference in top-10 candidate gene list precision scores are non-negative [except for motor subcortex (–), Fig. 7; calculation of precision values in Extended Data Fig. 7-1]. These results suggest that the method is valid and specific in identifying genes associated with memory and motor function.

## Discussion

Taken together, our results show that cortical and subcortical regions involved in human memory possess distinct genetic signatures. These genetic signatures are in agreement with prior research in animal models of memory, and were dissociable from the control of motor function. Thus, we show that the strong similarities between the spatial patterns of human brain transcriptome and the functional neuroimaging map of memory can be exploited to highlight candidate biological processes and genes associated with human memory for future experimental investigations. This may contribute to our knowledge of the functional differences of cortical and subcortical regions in healthy human memory function and memory disorders.

Presently, human memory evidence is generally derived from popular non-invasive methods such as GWAS (Wellcome Trust Case Control Consortium, 2007), which identifies links between gene variants and cognition (Heck et al., 2014). However, GWAS ignores the spatially distributed gene expression in the brain by solely analyzing gene variants in relation to brain or behavioral measures (Hawrylycz et al., 2012; Mahfouz et al., 2017). Our approach relies on spatial pattern of gene expression and identifies genetic profiles related to human memory. Crucially, our unsupervised approach is versatile as it can reveal unprecedented insights into any human cognitive function of interest, e.g., decision making. This insight may be especially useful in the case of functions that are clinically relevant but with a genetic basis that is less understood, e.g., attention (ADHD) and language (dyslexia).

To identify general human memory genes that function across the brain, we compared the differences and the overlap between cortical and subcortical memory genes (Fig. 1). Particularly, this overlap comparison is supported by the existence of genes underlying memory function as a whole, as in the case of neuronally-expressed immediate-early genes (IEGs) involved in memory function (Gallo et al., 2018). IEGs are a broad class of genes that are expressed in a rapid, transient manner in response to a plethora of cellular stimuli. Of the neuron-specific IEGs, *c-fos*, *Egr1*, and *arc* are broadly associated with various facets of memory across both cortical and subcortical areas. For example, the blockade of hippocampal *c-Fos* negatively impacted spatial long-term memory (Kemp et al., 2013), and its blockade in either the hippocampus or retrosplenial cortex induced deficits in the consolidation of fear memory (Katche et al., 2010; Katche and Medina, 2017). Such genes are relevant for different subtypes of memory across both cortical and subcortical areas, which we term whole-brain general memory genes.

If there are such general memory genes whose function in memory spans the whole brain, both cortical and subcortical analyses should show overlapping genes. We found that the cortical and subcortical areas possess largely distinct genetic profiles, as identified by gene-functional spatial correlation (Fig. 5). There was no overlap in the top-10 cortical and subcortical memory genes, with some overlap for memory genes (9.6% out of 1397 genes) and biological process gene sets (2.5% out of 118 gene sets).

At the biological process level, we found differences in cortical and subcortical memory. In the cortex, the identified gene sets included epigenetic regulation and immune signaling. The latter received recent interest as a central factor in the onset and progression of dementia (Litteljohn et al., 2014; Kim and Kaang, 2017; Hammond et al., 2019). In the subcortex, the identified genes are involved in neurogenesis and glial cell differentiation. Furthermore, we identified gene sets with a less understood link to memory as well. For instance, astrocytes and oligodendrocytes were recently discovered to be involved in linking glial-mediated potassium homeostasis and myelination to memory deficits (Hertz and Chen, 2016; Pepper et al., 2018). It is still unclear how myelinating oligodendrocytes may enable plasticity in memory (Pepper et al., 2018). Our work suggests that glial cell differentiation may play a complementary role in memory function, and should be further investigated for a comprehensive understanding of cellular contributions to memory. Overall, this may suggest inherent differences in the biological processes supporting cortical and subcortical memory regions. Future work may look into the interplay of these processes and clarify their differential contributions toward cortical and subcortical memory function.

At the gene level, enriched genes for cortical and subcortical memory were similarly distinct. Of the enriched genes that are associated with the biological processes above (in sets *S* ^+^ and *S* ^-^), a small proportion of genes (9.6%, or 135 genes) were shared between cortical and subcortical regions (Fig. 5). These genes are related to the Arp2/3 complex, GABA and AMPA ligand-gated ion channels, and srp-dependent protein localization to the membrane. The Arp2/3 complex is necessary for the maturation of dendritic spines, hippocampal and extra-hippocampal AMPA receptors are involved in excitatory ion channels in memory, and GABA receptor subunits are part of inhibitory ion channels in memory function (Collinson et al., 2002; Freudenberg et al., 2016; Spence et al., 2016). As such, this recapitulates known literature, and hints at basic requirements for general memory function. Overall, this may suggest differences due to gross cortico-subcortical differences in transcriptome profiles and function in healthy memory function and disease (Huber et al., 1986; Salmon and Filoteo, 2007). Future work may look into how the convergence and divergence between cortical and subcortical genetic profiles and how those enable cortico-subcortical-specific functions in memory.

Additionally, our approach also identified memory-associated genes with poorly understood relations to memory. For example, the *MIS18BP1* gene was identified in the subcortical memory genes (Extended Data Table 2-1). This gene is required for recruitment of centromere proteins to centromeres and allow normal chromosome segregation during mitosis (Moree et al., 2011). It is unclear whether such cell division genes play a role in memory across subcortical areas. However, the gene has been linked to hippocampal neurogenesis, which is critical for hippocampal function in memory (Shin et al., 2015; Gonçalves et al., 2016). Such lesser known genes constitute a crucial contribution of our framework, as their immediate link to memory yet to be established, and should be examined in future research.

Our analyses of gene expression and neuroimaging maps are not without limitations. These include the limited sample size, the validity of a text-mining-like approach with GSEA and Gene Ontology library, and the spatial resolution of the AHBA. First, the limited donor sample size and reduced genome coverage after preprocessing may contribute to a reduced power, but not statistical precision, of our approach. Although future increase in sample size may identify more genes using this method, we found the current results to be robust as our results are significantly better than chance (i.e., statistical significance). Furthermore, the identified genes were specific to memory, as demonstrated by the precision of our framework. Second, GSEA utilizes the Gene Ontology library to identify enriched gene sets, and associates these enriched genes with the library’s ontological terms, e.g., synaptic plasticity. We concede that the Gene Ontology library is continually being extended with manual curation efforts, and thus is vulnerable to being outpaced by the deluge of recent experimental findings (Baumgartner et al., 2007; Dutkowski et al., 2013; Gaudet and Dessimoz, 2017). As such, it is possible that the database is incomplete and does not reflect all biological functions associated with each gene. This may lead to false negatives, where we miss genes that should be considered enriched. Nevertheless, our approach demonstrates high effectiveness (as seen in the top-10 memory and motor function genes) and the results are in concordance with known experimental literature independent of ontology libraries. Additionally, unsupervised methods of identifying candidate genes always require manual curation and selection of these genes for further investigation. Third, this approach is also limited by the spatial resolution of the human brain transcriptome. Despite being the most appropriate human transcriptional atlas with its whole-genome and high resolution whole-brain coverage, the AHBA map still has a lower resolution compared to functional imaging maps, especially in the cortex (Hawrylycz et al., 2011). As such, we expect the precision and statistical power of our approach to grow as the spatial resolution and sample size of the AHBA database increases. Furthermore, as the translation of gene mRNA into a functional product is subject to regulation, donor brain proteomes may be complementary in identifying genes linked to memory (Lubec et al., 2003; Park et al., 2006; Sjöstedt et al., 2015).

## Conclusion

Here, using the Allen Institute brain transcriptional atlas and Neurosynth neuroimaging maps, we demonstrate that cortical and subcortical memory regions have distinct genetic signatures. These genetic signatures provide novel biological processes and molecular targets for understanding of human memory function. Crucially, we hope that our unsupervised and spatially guided approach may help guide researchers toward productive gene and biological process candidates for understanding how complex cognitive functions such as memory may be enabled by the molecular components of the brain.

## Synthesis

Reviewing Editor: Morgan Barense, University of Toronto Decisions are customarily a result of the Reviewing Editor and the peer reviewers coming together and discussing their recommendations until a consensus is reached. When revisions are invited, a fact-based synthesis statement explaining their decision and outlining what is needed to prepare a revision will be listed below. The following reviewer(s) agreed to reveal their identity: Anastasia Brovkin, Morgan Barense.

This article addresses the need for integrating micro-scale human brain gene expression and macro-scale human neuroimaging data to expand our understanding of the genetic underpinnings of neural correlates of human memory. This work illustrates an extension to the correlational analysis framework of Fox et. al, 2014 and claims to demonstrate that cortical and subcortical memory regions have distinct genetic signatures. While the use of this advanced statistical analysis/workflow is commendable, the manuscript needs major adjustments to effectively communicate its scientific contribution.

General comments:

1. The overall language of the article is organized and easy to follow, however the manuscript covers a lot of ground in the background review. This needs to be reworked to ensure audience can follow easily, for example with the use conceptual figures and clearer definitions, especially in the beginning.

2. The relationship between declarative vs. non-declarative memory as it relates to cortical vs. non-cortical neural substrates is not clear. This was made even less clear by the statement on page 8 that the hippocampus - a structure very closely associated with declarative memory - was labelled as a subcortical structure. We encourage the authors to abandon discussion of the declarative vs. non-declarative distinction, as recent work suggests that the brain does not honour this dichotomy (e.g., Cowell, Sadil, Barense, 2019, eNeuro). For example, the hippocampus appears necessary for processes that are conscious as well as non-conscious (e.g., statistical learning - see the work of Anna Schapiro and Nick Turk-Browne). There is simply not a 1:1 mapping between declarative/non-declarative and cortical/non-cortical.

3. Related to the above, the distinction between cortical vs. subcortical as it relates to cognition needs to be made clearer. First off, cortex is huge and many distinct cortical regions contribute to memory in very different ways (e.g., V1 vs. perirhinal cortex). How does this relate to the authors' questions and the fact that “cortical” regions are considered together?

4. A similar point could be made about the fact that the authors have considered “memory” as a single entity in the neurosynth classification. This means that all manner of memory (e.g., working memory, implicit memory, and long-term memory) would have been lumped together. While there is evidence that these types of memories share neural circuitry if they recruit the same underlying representation (e.g., implicit perceptual learning and recognition memory may both recruit the hippocampus if scene stimuli are involved; Graham et al., 2006 JoN, Taylor et al., 2007 Neuropsychologia), there are considerable differences between these memory functions and they would have different underlying mechanisms. This should be considered, and perhaps the authors may wish to focus their interpretation on differences between memory writ large vs. motor function.

5. The authors describe Alzheimer's disease as primarily affecting cortical structures, but much research highlights sub-cortical contributions to the disease - especially of the basal forebrain (see Schmitz et al., 2016, Nature Communications). This speaks to the larger issue that the distinction between cortical vs. subcortical contributions to cognition is not as dichotomous as presented by the authors.

6. We encourage the authors to state the core aim(s) or core hypothesis in a clearer fashion, in particular as it relates to the consideration of cortical vs. sub-cortical structures.

7. What is meant by “here we use a novel validated framework” - unclear - how was this framework “validated”?

8. It is not entirely clear why authors chose the AHBA transcriptome and Neurosynth dataset - why was the investigation limited to these data (i.e. other similar datasets, justification for chosen datasets)?

9. The transcriptomics dataset is limited - size and completeness of dataset is not sufficient to fully test hypothesis in exhaustive, empirical manner.

10. The scientific insight from this analysis is limited by several factors: reliability of Neurosynth mapping, errors in transformation to standard MNI space, delineation of cortical and subcortical areas, validity of datasets and text-mining “ontology -like” approach. Discussion of these limitations is warranted.

11. The abstract and title should be adjusted to emphasize that paper is more focused on providing an analysis framework. In fact, focusing on the contribution of the analysis framework, rather than distinctions between cortical/sub-cortical contributions to cognition may be a way to position the impact of the paper.

12. Related to the above, the phrasing of title is ambitious. The manuscript presents data mining and validation framework but no novel, empirical insight can be extracted from this investigation alone - at least going by the discussion and conclusion sections as is

13. It is not fully clear why the authors chose to rank the gene lists and what this means for interpretation. (What do performance criteria mean?)

Minor concerns:

1. Which atlas was used to delineate cortical and subcortical regions - this is not fully apparent from the processing the transcriptome section - authors may also wish to justify such methodological choices

2. Figure 2 could be improved to visualize the spatial similarity data - an alternative choice of graph or different scale may aid visualization better - or even an interactive graph provided in external data section

3. There were some minor errors in the citations: Article cited as (Atzmon et al, 2018) could only be found as Freudenberg-Hua, Y., Li, W., & Davies, P. (2018). The role of genetics in advancing precision medicine for Alzheimer's disease-A narrative review. Frontiers in medicine, 5, 108.

4. Funding information should be provided.

5. The authors should be careful with broad claims such as “identifying drug candidates for memory disorders” since the work is correlational in nature.

6. We encourage the authors to be careful with “causal language” - i.e. “neural correlates”, “genetic underpinnings” - this is often too vague to carry interpretability and may become a target for criticism

7. The visual abstract is informative as it relates to the analysis pipeline in the Results section. Figure is informative - with regards to the analysis steps - but missing scientific conclusion/ summary of end results - in this case it is difficult to extract conclusive results of analysis and how these support the hypothesis
