# Supplementary figures and images for: Distinct Genetic Signatures of Cortical and Subcortical Regions Associated with Human Memory
Source: eNeuro. 2019 Dec 13;6(6):ENEURO.0283-19.2019. doi: 10.1523/ENEURO.0283-19.2019 (PMC6917897; doi:10.1523/ENEURO.0283-19.2019)

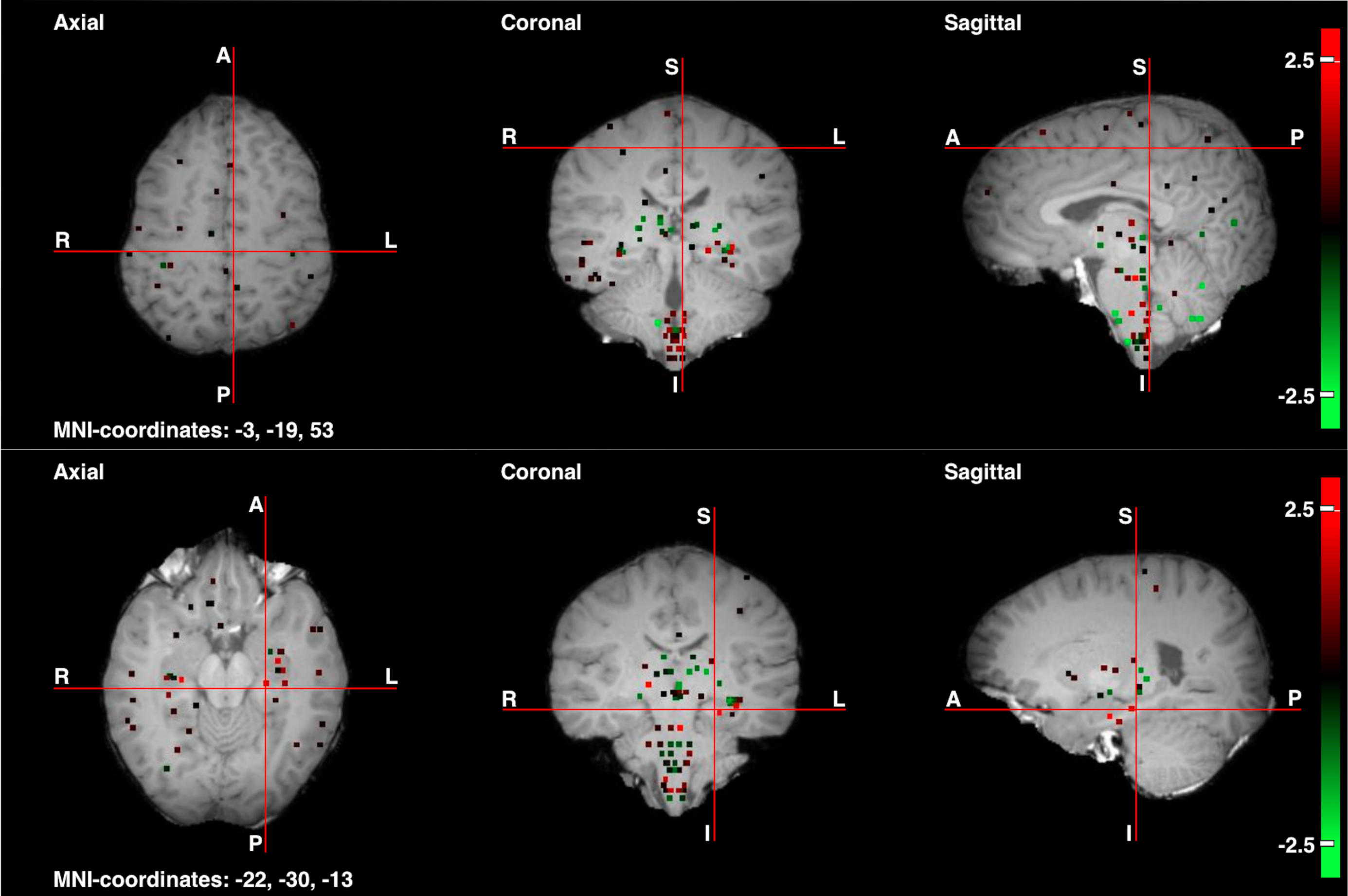

Supplement: Extended Data Figure 1-1 — Visualization of the GRB14 gene expression from the AHBA. Visualized Allen human brain transcriptome atlas on a single donor showing the gene expression z score of regions involved in memory and motor function. Created with the planar viewer at http://human.brain-map.org/. Download Extended Data Figure 1-1, TIF file. [file sup_enu-eN-NWR-0283-19-s02.tif]

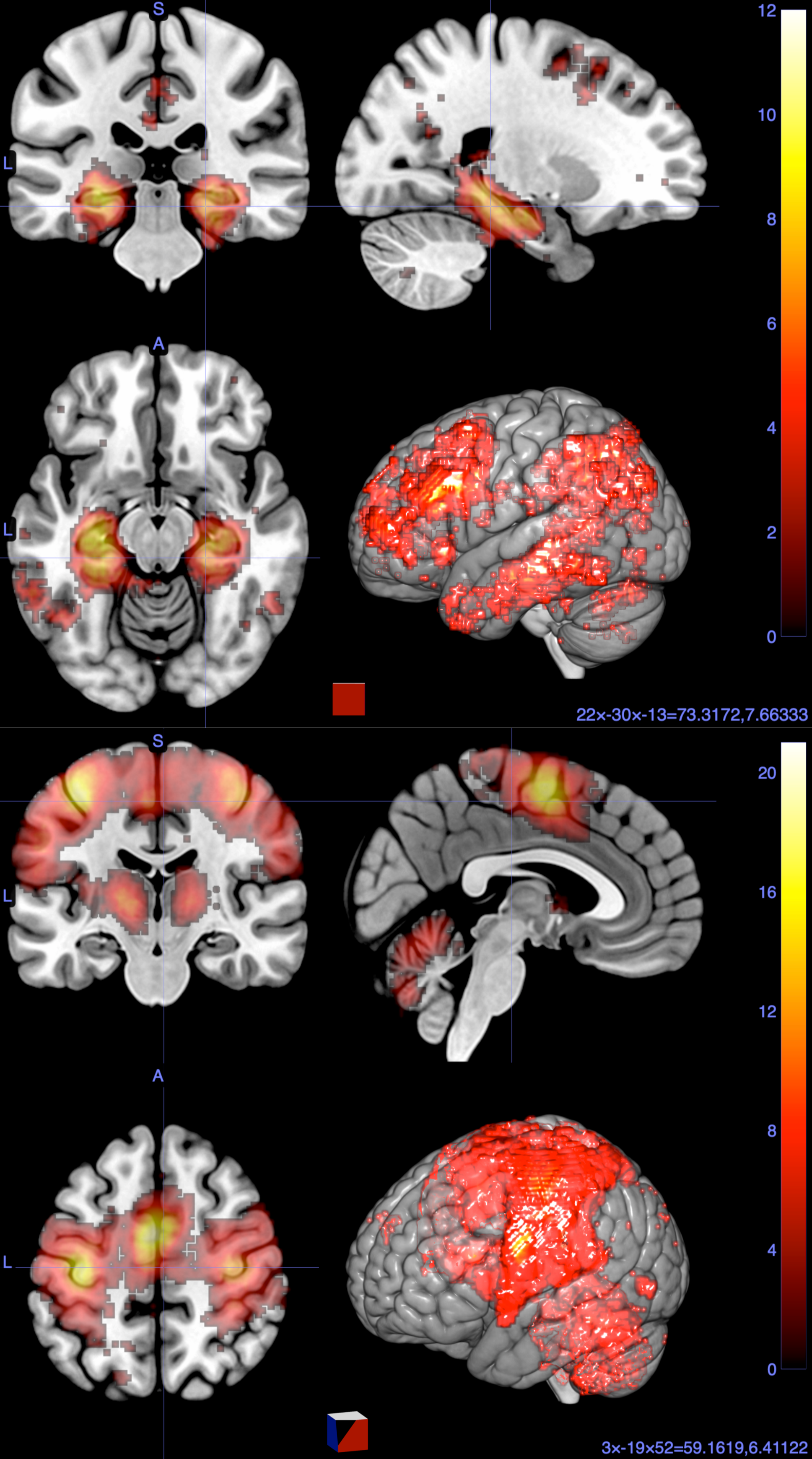

Supplement: Extended Data Figure 1-2 — Visualization of Neurosynth memory and motor neuroimaging maps. Visualized Neurosynth maps using MRIcro showing the z score intensity of regions involved in memory and motor function. Only positive z scores are used. Download Extended Data Figure 1-2, TIF file. [file sup_enu-eN-NWR-0283-19-s03.tif]
